# “Hidden” Voices Marginalised community perspectives on policing and community safety; an international scoping systematic review

**DOI:** 10.1192/j.eurpsy.2025.804

**Published:** 2025-08-26

**Authors:** L. Jordan, G. Gulati, C. Dunne, B. Kelly, M. Donnelly

**Affiliations:** 1 School of Medicine; 2 School of Law, University College Cork, Cork; 3 School of Medicine, University of Limeirck, Limerick; 4 School of Medicine, Trinity College Dublin, Dublin, Ireland

## Abstract

**Introduction:**

Community safety is about everyone having the right to be and feel safe in their community. People from marginalised communities (including people with mental illness, intellectual disability, migrants, and homeless) are over-represented in policing contacts. Yet, little is known about the real world perspectives of these marginalised groups in respect of perceived safety and interventions that work to improve this.

**Objectives:**

To systematically review the published literature concerning the experiences of people from marginalised communities on policing and community safety.

**Methods:**

Research database SCOPUS (inception to 1 January 2024) was searched for English-language publications using key words. The electronic search was augmented by manual searching through reference lists and websites of governmental and non-governmental organisations. Published studies with information about the experiences of persons from marginalised communities on policing and community safety were included. Opinion articles or reviews that did not contain qualitative data were excluded, as were studies that focused on law enforcement professionals views.

**Results:**

Of the 857 papers identified, 17 studies met eligibility criteria with a total of 1254 participants from 5 countries. A recurring theme from different marginalised communities was “greater fear” and “less trust” of police and a reluctance to report crime. Those with physical disabilities were less likely to use public transport. Latin migrants feared speaking Spanish in America. African refugees in Australia felt targeted by the police because of their ethnicity. Muslims in England reported they were under increased police surveillance. Homeless youths in Canada with early negative experiences with law enforcement personnel were less likely to seek police involvement if needed in future. Conversely both Mexican-origin residents and Chinese immigrants living in America identified police as having a critical role in making them feel safe.

**Conclusions:**

This study scoped the experiences of people from marginalised communities in respect of policing and community safety. To the author’s knowledge, this is the largest scoping study of this type, to date. It is evident from this review that there are voices, sometimes “hidden voices”, from marginalised communities that perceive policing approaches differently. This guides not only their future interactions with police but also their social outlook. Working closely and proactively with individuals within these marginalised communities will help find the balance between “over policing” and “under policing” to help contribute to the overall community safety. A key recommendation from this review would be for authorities to meaningfully incorporate these voices when developing or reviewing policies relating to community safety.

**Disclosure of Interest:**

None Declared

